# Morphine for the Treatment of Pain in Sickle Cell Disease

**DOI:** 10.1155/2015/540154

**Published:** 2015-01-12

**Authors:** Mihir Gupta, Lilian Msambichaka, Samir K. Ballas, Kalpna Gupta

**Affiliations:** ^1^Stanford University School of Medicine, Stanford, CA 94305, USA; ^2^Vascular Biology Center, Division of Hematology, Oncology and Transplantation, Department of Medicine, University of Minnesota Medical School, Mayo Mail Code 480, 420 Delaware Street SE, Minneapolis, MN 55455, USA; ^3^Christiana Care Health System, Department of Internal Medicine & Pediatrics, Newark, DE 19713, USA; ^4^Cardeza Foundation for Hematologic Research, Jefferson Medical College, Thomas Jefferson University, Philadelphia, PA 19107, USA

## Abstract

Pain is a hallmark of sickle cell disease (SCD) and its treatment remains challenging. Opioids are the major family of analgesics that are commonly used for treating severe pain. However, these are not always effective and are associated with the liabilities of their own. The pharmacology and multiorgan side effects of opioids are rapidly emerging areas of investigation, but there remains a scarcity of clinical studies. Due to opioid-induced endothelial-, mast cell-, renal mesangial-, and epithelial-cell-specific effects and proinflammatory as well as growth influencing signaling, it is likely that when used for analgesia, opioids may have organ specific pathological effects. Experimental and clinical studies, even though extremely few, suggest that opioids may exacerbate existent organ damage and also stimulate pathologies of their own. Because of the recurrent and/or chronic use of large doses of opioids in SCD, it is critical to evaluate the role and contribution of opioids in many complications of SCD. The aim of this review is to initiate inquiry to develop strategies that may prevent the inadvertent effect of opioids on organ function in SCD, should it occur, without compromising analgesia.

## 1. Introduction and Background

Sickle cell disease (SCD) continues to afflict millions of people worldwide and the disease is on the rise [1]. Pain is a hallmark feature of SCD that can begin in infancy and increase in severity throughout life. Severe pain is the most common clinical manifestation of SCD, leading to hospitalization, opioid consumption, and increased risk of shorter survival [2]. However, investigation on pain and its treatment in SCD remained underexplored until recently. An area that still remains unaddressed is the consequence of frequent high doses of opioids in sickle patients. On the whole, side effects of opioids are poorly understood and opioid-induced hyperalgesia (OIH) is beginning to be appreciated.

Long-term opioid use is associated with undesirable consequences including physiologic tolerance, hyperalgesia, and respiratory depression [3–5]. Available data suggest that opioids influence vascular [6], pulmonary [7, 8], and renal function [9, 10] and cancer progression [6]. Our group (Gupta et al.) found that higher opioid requirement was independently associated with shorter survival in patients with advanced prostate cancer [11] and lung cancer [12]. However, it remains to be determined whether high opioid use is a cause or consequence of this phenomenon. Heroin, which metabolizes to morphine* in vivo*, is associated with nephropathy in humans [13]. Because of shorter lifespan and multiorgan complications including renal, pulmonary, and vascular function in SCD, it is critical to understand if opioids exacerbate organ damage in SCD and concurrently introduce serious complications and comorbidities of their own. We review the critical findings on opioid-induced adverse effects and associations of opioid use in experimental and clinical studies. Because of lack of experimental and clinical data on opioid side effects, this review is intended to raise awareness of this issue so that experimental and clinical studies can be undertaken in near future.

## 2. Complex Pathophysiology of SCD Is Intertwined with Pain

In SCD, clustering of sickle red blood cells (RBCs) leads to vascular occlusion, impairment of oxygen supply to the tissues leading to organ damage, and acute painful episodes called vasoocclusive crises (VOCs) [2, 14]. Characterized by enormous complexity and phenotypic variability, SCD is associated with unpredictable, recurrent, and acute VOC, in addition to chronic pain and ischaemic organ damage [2, 14]. Pain can begin in infancy and recur through adult life, causing frequent hospitalizations, impairment of quality of life, and reducing survival [2, 14, 15]. Some sickle pain can be acute, recurrent, persistent, chronic, or mixed and could be due at least in part to OIH. The lifelong, progressive nature of pain in SCD necessitates chronic opioid use, resulting in suboptimal analgesia and contributing to poor quality of life [2]. Patients with SCD often remain undertreated due to opioid inefficacy and providers' fear (opioid phobia) of addiction potential. Conversely, some patients may be overtreated because of subjective measures of pain. A recent study to evaluate pain and opioid treatment in SCD had to be concluded prematurely because of poor enrollment and multiple challenges [16]. However, it is known that both acute and chronic pain require relatively higher doses of opioids for longer durations in SCD than in many other chronic pain conditions [2, 17, 18]. Clearance of morphine was found to be almost twofold in sickle patients as compared to normal subjects, which argue for the higher opioid dose administration to achieve pharmacologically therapeutic levels [19, 20].

Recent studies in Berkley sickle mice have provided critical insights into the pathobiology of pain. The skin of these mice shows abnormal peripheral nerve fiber architecture, which may underlie the observed activation of nociceptors and increased hyperalgesia [21]. Indeed, TRPV1 channels were activated on the nociceptors of peripheral nerve fiber in these sickle mice [21, 22]. These mice also exhibit characteristics of pain observed in SCD, including musculoskeletal pain and increased sensitivity to mechanical, heat, and cold stimuli, which are further exacerbated by hypoxia/reoxygenation (that simulates VOC) [21, 23]. Peripheral nociceptor activation appears to be mediated by mast cell activation and neurogenic inflammation because treatment of sickle mice with the mast cell inhibitor imatinib reduced neurogenic inflammation and hyperalgesia and reduced the requirement of morphine dose [24]. It is hypothesized that mast cell activation results in release of inflammatory cytokines and neuropeptides, which promote nociceptor activation and enhance neuropeptide release from peripheral nerve terminals, thus contributing to continued pain. The mechanism of mast cell's role in sustained hyperalgesia is based on the studies in sickle mice. However, two separate clinical studies on sickle patients show that the use of imatinib, a known mast cell inhibitor, significantly reduced painful episodes in patients with SCD [25, 26]. Another case report showed that a sickle patient who died following an overdose of fentanyl was on fentanyl for more than 18 months and had pruritis and sickle crises type of pain for 2 days as well as ACS and respiratory depression secondary to fentanyl overdose [27]. His blood showed significantly high blood concentration of mast cell tryptase (76 microg/L as compared to the normal value of 2–4 microg/L). These correlative mouse and human studies suggest that therapies based on the inhibition of mast cell activity need to be investigated in a larger clinical trial in sickle patients.

## 3. Molecular and Cellular Effects of Opioids

### 3.1. Pharmacological Aspects of Opioids and Opioid Receptors Relevant to SCD

Morphine is absorbed from the gastrointestinal tract [28, 29] and metabolized in the liver, gastrointestinal tract, and kidneys [30]. The major pathway for the metabolism of morphine is conjugation with glucuronic acid and liver is the main metabolic site but it can also be metabolized in the brain and kidney [28]. Elimination is through bile or urine [31]. Morphine-3-glucuronide (M3G) and morphine-6-glucuronide (M6G) are the major metabolites [28]. M6G has pharmacological actions that are indistinguishable from morphine. The interaction with opioid receptors by the glucuronides may thus contribute to the pharmacological and/or toxicological effects of morphine. M3G has no analgesic effects but may cause some of the side effects of morphine.

Morphine and its congeners (hydromorphone, fentanyl, etc.) act via G-protein coupled opioid receptors (ORs) [32]. Four different ORs have been identified, namely, mu-, delta-, kappa-, and nociceptin-OR (MOR, DOR, KOR, and NOP/OR, resp.), but the analgesic activity of opioids is mediated via the MOR. ORs undergo phosphorylation by G-protein coupled receptor kinases and subsequent *β*-arrestin recruitment, thus uncoupling the receptor from its G protein, followed by endocytosis, degradation, and downregulation [33]. However, MORs are recycled back to the cell membrane following endocytosis. The “net signal” for relative activity of the receptor versus endocytosis, termed “relative activity versus endocytosis” (RAVE), is the ability of an opioid agonist to induce signaling and to promote endocytosis. Morphine has a high RAVE value as a consequence of its* inability* to promote receptor desensitization and endocytosis [34]. Additionally, MOR can be constitutively activated and/or can display elevated constitutive activity following prolonged agonist treatment [33, 35]. The implication is that short-term, repeated, or chronic morphine treatment may lead to sustained effects on target tissues. Therefore, recurrent opioid use during VOC may lead to continued opioid activity in the target tissues in the intermittent period between VOC episodes and/or after opioids are discontinued.

### 3.2. Molecular and Cellular Effects of Opioids That May Influence SCD Pathophysiology

**Figure 1:**
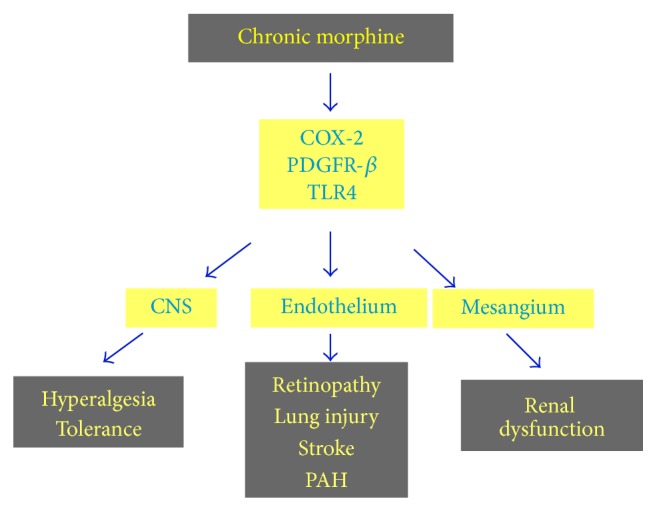
Proposed model of morphine-induced signaling leading to organ damage. Morphine signaling via cyclooxygenase-2 (COX-2), platelet-derived growth factor-β (PDGFR-β), and toll-like receptor 4 (TLR4) may underlie the morphine-induced hyperalgesia and tolerance via its action on the central nervous system (CNS); promote endothelial dysfunction and associated retinopathy, lung injury, pulmonary arterial hypertension, and stroke; and contribute to renal dysfunction in sickle cell disease.

In addition to analgesia induction, opioids activate growth, survival, and cytoprotection via opioid receptors in multiple cell types in the peripheral organs and in the central nervous system [6, 36]. Morphine stimulates diverse neural and nonneural molecular targets. Morphine induces expression of platelet-derived growth factor-BB (PDGF-BB) in human brain- and umbilical vein-endothelial cells and PDGF receptor-*β* (PDGFR-*β*) expression in pericytes and increases vascular permeability [37, 38]. Morphine also transactivates receptor tyrosine kinases (RTKs) for vascular endothelial growth factor receptor-2 (VEGFR2), PDGFR-*β*, sphingosine 1 phosphate receptor 3 (S1P3R), mitogen activated protein kinase/extracellular signal related kinase (MAPK/ERK), and cyclooxygenase-2 (COX-2) in endothelial cells and the central nervous system [6, 39–42]. Several cytokines including PDGF and VEGF that stimulate RTKs are elevated in SCD [43–45]. Morphine stimulates the expression of PDGF-BB in endothelial cells, known to increase vascular permeability [38]. Levels of several cytokines including PDGF and VEGF that stimulate RTKs are elevated in patients with SCD [45]. Thus, morphine administration may amplify endothelial activation and promote organ dysfunction such as retinopathy, strokes, and pulmonary hypertension, in SCD, as discussed below (Figure 1).

### 3.3. Activation of TLR4

Morphine binds myeloid differentiation protein-2 (MD-2) inducing toll-like receptor-4 (TLR4)/MD-2 oligomerization required for TLR4 signaling [46]. Independent of opioid receptors, morphine can induce inflammation and potentiate hyperalgesia in rodents via TLR4 [47]. Our group found that TLR4 expression is increased in the spinal cord and cutaneous mast cells of mice expressing human sickle hemoglobin as compared to control mice [21, 24]. Morphine treatment* in vitro* leads to the activation of cutaneous mast cells from control and sickle mice and* in vivo* in breast tumors in mice, leading to the release of inflammatory cytokines and neuropeptides, substance P (SP), and calcitonin-gene related peptide (CGRP) [24]. In sickle mice activation of TLR4 underlies vasoocclusion and acute lung injury [48, 49]. Increased levels of neuropeptide SP were described in sickle patients at steady state, which increased further during VOC [50]. Pain was not evaluated in this study. It is possible that use of opioids during VOC contributed to an increase in SP. Thus, while providing analgesia via MOR, morphine may simultaneously play a detrimental role in SCD by promoting neuroinflammation, vascular dysfunction, and hyperalgesia via TLR4 activation. These experimental data argue for examining the cotreatment strategies of inhibition of TLR4 with morphine and evaluate the contribution of opioids to the exaggeration of inflammatory and neuroinflammatory microenvironment in SCD.

## 4. Implications of Opioid Exposure for Organ Dysfunction in SCD

### 4.1. Renal Disease

Renal complications that start early in age and may progress to end-stage renal disease (ESRD) are a leading cause of morbidity and mortality in adults with SCD [51, 52]. Survival is estimated to be 4 years following the onset of ESRD even when receiving dialysis. The pathophysiology of sickle nephropathy is not clearly understood but it involves both glomerular and tubular injury accompanied by proteinuria, hyperfiltration, increased glomerular filteration rate (GFR), blood flow and tubular resorption, and glomerulosclerosis. Renal microenvironment in SCD is attended by oxidative stress, iron deposition, ischemia/reperfusion injury and pulmonary hypertension, and altered hemodynamics with increased hemoxygenase-1 and COX-2 and reduced NO bioavailability. Early renal disease includes glomerular hyperfiltration, increased proximal tubular function, and hematuria. Subsequently the concentrating ability is reduced; there is focal segmental glomerulosclerosis with proteinuria, papillary necrosis, and reduced glomerular filtration [53].

### 4.2. Influence of Opioids on Renal Disease

**Figure 2:**
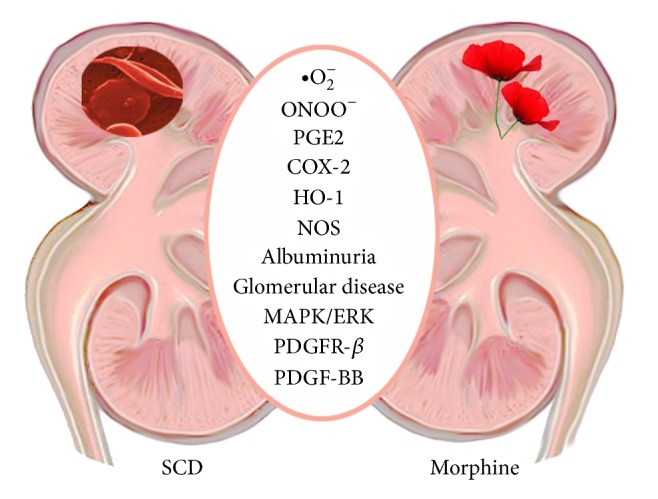
Proposed model of morphine activity in the kidney. Morphine stimulates cyclooxygenase-2 (COX-2), hemoxygenase-1 (HO-1), inducible/endothelial nitric oxide synthase (i/e NOS), oxidative stress via reactive oxygen species/peroxynitrite, mitogen activated protein kinase/extracellular signal regulated kinase (MAPK/ERK), platelet-derived growth factor receptor-β (PDGFR-β), and platelet-derived growth factor-BB (PDGF-BB). These molecular changes are accompanied by albuminuria and glomerular pathology in morphine treated mice. Together, these morphine-induced cellular, molecular, and pathological effects may stimulate and exacerbate existent renal damage in sickle cell disease.

Clinical and experimental studies have demonstrated the toxic effects of the chronic use of opioids on the kidney. We and others have observed that clinical doses of morphine and hydromorphone incite kidney pathology, glomerular enlargement, and albuminuria in wild type and transgenic sickle mice [10, 54, 55]. Opioids cause renal damage as evidenced by renal tubular vacuolization, mononuclear cell infiltration, and focal necrosis in rats receiving morphine or levo-alpha-noracetylmethadol, a metabolite of levo-alpha-acetylmethadol, a long-acting MOR agonist [56, 57]. Morphine and opioid peptides have direct effects on mesangial and glomerular epithelial cells, kidney fibroblast, and the interaction of mesangial cells with circulating macrophages and PMNs via the production of superoxide [58, 59]. Through this interaction, morphine has the potential to directly impair slit diaphragm cell membranes in podocytes, contributing to kidney injury. Morphine-induced generation of reactive oxygen species (ROS) and production of superoxide by macrophages and mesangial cells induce podocyte DNA damage [58]. Morphine-induced podocyte injury leads to albuminuria in wild type mice [10]. Morphine treatment led to albuminuria and podocyte injury as well as diminished expression of podocyte markers, synaptopodin, and nephrin, in wild type FVBN mice [10], and increased podocyte foot process effacement accompanied by albuminuria in sickle mice [60]. Morphine stimulates proliferation of glomerular mesangial cells [9] and superoxide production [58], enhances deposition of ferritin-antiferritin complexes in the glomerulus [61], amplifies nitrite production [62], and stimulates COX-2 in the kidneys of mice treated with morphine [54]. Morphine amplifies renal pathology, stimulates albuminuria, and impairs renal function, in sickle mice, which share the disease phenotype with humans [55]. Therefore, morphine treatment may stimulate and/or further augment renal injury (Figure 2).

Sickle and control mice treated with morphine demonstrate increased phosphorylation of PDGFR-*β* and MAPK/ERK and glomerular cell marker Thy-1 in the kidneys as compared to PBS. PDGFR-*β*, MAPK/ERK, and Stat3 signaling pathways play a central role in kidney disease. We (Gupta et al.) observed that morphine-induced mesangial proliferation is dependent on PDGFR-*β* and Stat3 signaling via MOR and KOR [55] and accompanied by increased kidney weight and glomerular volume expansion in wild type and sickle mice [9, 54, 60]. Since morphine also leads to PDGF-BB expression in endothelial cells, it is likely to amplify PDGFR-*β* signaling by direct coactivation of the receptor and also via the release of PDGF-BB in the kidney. Higher expression of MOR and KOR in sickle mouse kidneys may further augment the activity of morphine manifested as renal dysfunction demonstrated by proteinuria, higher BUN, and reduced BUN clearance in sickle mice and increased BUN in Wistar rats following chronic morphine treatment [55, 56]. Morphine-induced tubular damage observed in mice and rats [56, 60] may additionally contribute to renal dysfunction. Increased PDGF-BB levels have been reported in sickle patients as compared to normal subjects [45]. Pain and opioid use were not evaluated in this study. No human data could be found on the effect/association of opioid use with nephropathy in SCD. However, heroin-associated nephropathy was recognized in chronic drug users more than three decades ago [63] but the possibility of a similar nephropathy in chronic morphine (a metabolite of heroin) users remains unexamined. Moreover, intravenous opiate addiction has been considered a risk factor for the development of human immunodeficiency virus (HIV) associated nephropathy [56, 58]. Data suggest both central and sympathetic nervous system dependent and independent effects of opioids on renal function [64]. Therefore, whether morphine contributes to sickle nephropathy in humans merits careful examination.

### 4.3. Pulmonary Disease

Pulmonary disease is another major cause of morbidity and mortality in adults with SCD but its etiology is not well understood. In sickle patients, morphine is associated with an increased risk of developing acute chest syndrome (ACS) [7, 65]. In a retrospective analysis of children with SCD, the frequency of ACS was significantly higher in the morphine treated group (29%) as compared to 12% in those treated with Nubain, a synthetic opioid antagonist/agonist related to naloxone and oxymorphone [7]. Causes of ACS include pneumonia, bone marrow fat embolism, pulmonary infarct due to* in situ* sickling, rib/sternal infarction, infection, and pulmonary embolism (PE) [18, 66–68]. Approximately 50% of patients with ACS have no identifiable etiology [18, 69]. Acute chest syndrome is closely associated with VOCs, especially in adults [18, 69, 70]. It occurs in approximately 50% of hospitalized patients with SS for VOC [18, 69, 71–73]. These hospitalized patients were given opioids, mostly morphine for pain management. This sequence of events suggests that opioids including morphine may have been instrumental in causing ACS especially in the 50% of patients on whom no identifiable cause was found. This is further supported by three observational reports showing that the use of morphine in patients with SCD seems to be associated with acute chest syndrome [7, 74, 75].

As discussed above, morphine stimulates TLR4 activity, and TLR4 has been implicated in acute lung injury and vasoocclusion in sickle mice [48, 49]. Moreover, the patient discussed above who died of fentanyl toxicity may have had fentanyl-related ACS, although the autopsy findings were not entirely convincing [27]. The association of morphine with increased frequency of ACS, therefore, merits further investigation.

### 4.4. Pulmonary Arterial Hypertension (PAH)

PAH is a major cause of morbidity and mortality in adults with SCD and may be associated with ESRD [76]. Right heart catheterization is the gold standard for the diagnosis of pulmonary hypertension [77, 78]. It is well known that people with SCD are at increased risk of PAH and PAH is a poor prognostic indicator. Yet, the pathogenesis of pulmonary hypertension in patients with SCD is not known. Several mechanisms have been proposed including hemolysis leading to nitric oxide (NO) deficiency, interstitial fibrosis secondary to ACS and vasculopathy characterized by endothelial dysfunction, increased vascular tone, inflammation, hypercoagulability, and vascular remodeling and destruction of pulmonary vasculature [79–81]. Increased plasma PDGF-BB concentrations were associated with increased odds of TRV in patients with SCD [45]. Morphine stimulates PDGF-BB expression in human brain- and umbilical vein-endothelial cells [37, 38]. Morphine may therefore influence PAH by augmenting PDGF-BB concentration.

In pulmonary hypertension, the initial apoptotic injury of pulmonary endothelial cells followed by hyperproliferation of apoptosis-resistant cells is believed to be one of the causes. Morphine has been implicated in simian immunodeficiency virus- (SIV-) induced PAH. Morphine treatment led to pulmonary vascular remodeling caused by enhanced apoptosis and endothelial proliferation in SIV-infected macaques [8]. It is noteworthy that morphine did not stimulate vascular remodeling in uninfected macaques. This clearly indicates that vascular responsiveness to morphine is distinct in a proinflammatory microenvironment, as compared to normal conditions. Similarly, in a tumor microenvironment replete with inflammatory cytokines, morphine promotes angiogenesis [6, 82]. It is therefore possible that the vasculopathic effects of morphine may contribute to development of PAH in an inflammatory microenvironment encountered in SCD.

Hemin-induced acute lung injury in sickle mice is mediated by TLR4 [49]. Since morphine activates TLR4 signaling, it may induce the pulmonary complications seen in SCD. Endothelial TLR4 signaling is also associated with hemolysis-induced VOC in sickle mice [48]. TLR4 gene expression is upregulated severalfold in cutaneous mast cells from sickle mice as compared to control mice. Morphine activated the release of tryptase and neuropeptides from mast cells from both control and sickle mice [24]. Mast cell proliferation and activation may contribute to PAH in humans [83]. In sickle mice, mast cell inhibitors reduce inflammation and improve morphine analgesia [24]. Thus, whether morphine may contribute to PAH in SCD via activation of TLR4 and mast cells is unknown at present. These hypotheses are speculative, but emerging mechanisms of morphine's role in vascular biology and the known role of vascular dysfunction and inflammation in sickle pathobiology provide a compelling rationale to pursue experimental and clinical studies to evaluate the role of morphine in PAH.

### 4.5. Other Organ Systems

We speculate that the activity of morphine on the vasculature may exacerbate preexisting endothelial vasculopathy and multiorgan dysfunction leading to devastating complications such as retinopathy and cerebral strokes in SCD. Proangiogenic signaling and angiogenesis stimulated by morphine may promote proliferative sickle retinopathy and collateralization in ischemic strokes, while morphine-induced vascular permeability may contribute to hemorrhagic strokes in SCD. Morphine use was associated with a 4.24- and 2.90-fold higher risk of hemorrhagic and ischemic stroke in prostate cancer patients and the risk increased with increase in morphine dosage [84].

Morphine-induced pruritis is another common feature in SCD patients [85]. Significantly less itching was observed with controlled release oxycodones as compared to controlled release morphine in cancer patients with pain [86]. The contribution of mast cell activation described above, therefore, deserves consideration in morphine-induced pruritis.

Opioid-induced clinical manifestations of the gastrointestinal system have been well known. Opioid-induced constipation (OIC) is reported in almost 35–70% of patients using opioids (including morphine, oxycodone, fentanyl, and others) [87, 88]. A peripherally acting OR antagonist with high affinity to MOR has been shown to reduce opioid-induced OIC [89]. Another side effect induced by opioid analgesia in patients is vomiting, which persists upon intrathecal delivery as well [90, 91]. Both constipation and nausea appear to be mediated by MOR in the GI as well as the CNS. Therefore, careful usage of MOR antagonists needs to be explored for these side effects while using opioids.

### 4.6. Effect on Red Blood Cells

Morphine directly diminishes normal RBC deformability in rats with morphine dependence [92]. Morphine treatment led to a decrease in RBC membrane fluidity and alterations in the secondary structure of membrane proteins. This would be expected to further exacerbate any problems with microvascular flow for sickle RBCs. We observed increased vascular congestion in kidneys of morphine treated sickle and wild type mice [54, 60], possibly due in part to a morphine-induced alteration in the rheological properties of RBCs. Increased iron deposits have been reported in the kidney of sickle patients by magnetic resonance imaging [93]. The mechanism by which iron is deposited is unclear, but it is almost certainly due to filtered iron from intravascular hemolysis [93]. Complementary to these* in vitro* and experimental observations, opioid drugs have been shown to influence whole blood rheology and cause morphometric and hematometric alterations in erythrocytes in drug users [94], including high incidence of anemia amongst heroin users [95]. MOR is expressed on human RBCs and its expression is increased in chronic opioid users [96]. In this study, RBCs with increased MOR expression also showed higher deformability indices and dehydration. Additionally morphine inhibited the activity of glutathione reductase purified from human erythrocytes* in vitro *[97]. This may in turn exacerbate existent oxidative stress, existent in SCD.

### 4.7. Therapeutic Benefit of Topical Morphine Treatment in Leg Ulcers in SCD

Like pain, leg ulcers in SCD are a debilitating condition causing more pain and contribute to the poor quality of life [18, 98]. This has been a neglected complication, which has recently gained attention from several groups worldwide [98–103]. Since morphine promotes angiogenesis, it can promote healing. We found that topically applied MOR agonist opioids, morphine, hydromorphone, and fentanyl accelerated closure of ischaemic open wounds in normal Fischer 344 rats [104]. Similarly, in leptin receptor mutant Zucker diabetic fatty rats, fentanyl accelerated wound closure as compared to PBS treated wounds [105]. Morphine stimulated angiogenesis, lymphangiogenesis, and nerve fiber density in the wounds and increased endothelial and inducible nitric oxide synthase, NO, and phosphorylation of PDGFR-*β* [104, 105]. Pain was not examined in these studies. On the basis of several clinical studies, a comprehensive review described that opioids applied topically significantly reduced pain in chronic wounds due to multiple pathologies, without any adverse effects [106]. However, when opioids were given systemically by other routes such as subcutaneous, intravenous, or orally, they did not ameliorate wound pain. Similarly, in our studies on Fischer 344 rats, morphine delivered via osmotic pumps implanted subcutaneously away from the wound site did not have any effect on wound closure, but topically applied opioids on the wound accelerated closure [104]. MOR signaling has been shown to heal the intestinal injury in mice [107]. Deletion of MOR resulted in thinner epidermis in mice [108]. Previous studies from our laboratory showed that sickle mice have significantly thinner epidermis and reduced MOR expression in the skin as compared to control mice [21]. Thus, examination of the opioid/opioid receptor system and therapeutic potential of topically applied opioids to reduce pain and promote healing of leg ulcers in SCD deserves consideration.

## 5. Conclusions and Future Directions

Overall the side effects of opioids remain poorly defined in clinical studies with a few exceptions. There are no controlled trials to compare the safety and efficacy of different opioids in the management of acute sickle cell crises. Patient safety can be maximized by obtaining a detailed history; understanding opioid pharmacology, mechanism of action, and side effects; carefully monitoring patients; and individualizing care.

**Figure 3:**
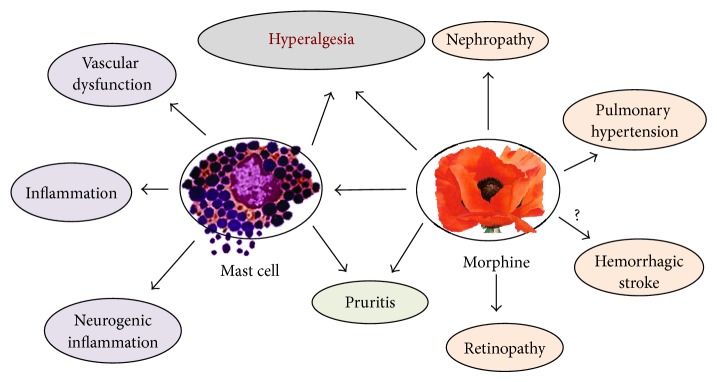
Proposed model of mast cell mediated as well as direct effects of morphine on vascular dysfunction and organ damage. Directly as well as via mast cell activation morphine may augment vascular dysfunction and inflammation. Through its multicellular and organ specific activities, morphine may influence hyperalgesia (pain), retinopathy, pruritis, stroke, pulmonary arterial hypertension, and nephropathy in SCD.

*In vitro* and preclinical studies raise awareness about the possible adverse and/or beneficial effects of opioids in the pathophysiological setting of SCD (Figure 3). Therefore, simultaneous strategies to ameliorate the adverse side effects need consideration. Cannabinoids have shown efficacy in treating chronic, inflammatory, and hypoxia/reoxygenation-induced acute pain in sickle mice [21, 23], offering an alternative (or adjunct) to opioid treatment if their efficacy is confirmed in human trials. Targeting TLR4 or mast cells offers the advantage of reducing pain and bypassing morphine tolerance. Clinically available drugs such as imatinib may target several key mechanisms including inhibition of PDGFR-*β* and mast cells as well as reduction in morphine tolerance [24, 55]. Another strategy may be coadministration of COX-2 inhibitors which may have an opioid sparing effect [109] and simultaneously inhibit the adverse effects of opioids on renal hemodynamics. Opioid-induced peripheral effects can also be antagonized by coadministration of peripherally selective opioid receptor antagonists [110]. Before advancing to clinical use, however, newer strategies need to be tested for potential adverse effects on the pathophysiology of SCD using validated transgenic mouse models of SCD.

## Abbreviations

ACS: Acute chest syndrome
COX-2: Cyclooxygenase-2
DOR: Delta opioid receptor
ESRD: End-stage renal disease
GAG: Guanine-adenine-guanine
GTG: Guanine-thymine-guanine
HbS: Sickle hemoglobin
GFR: Glomerular filteration rate
HO-1: Hemoxygenase-1
KOR: Kappa opioid receptor
M3G: Morphine-3-glucuronide
M6G: Morphine-6-glucuronide
MAPK/ERK: Mitogen activated protein kinase/extracellular signal regulated kinase
MOR: Mu opioid receptor
NO: Nitric oxide
NOS: Nitric oxide synthase
OIH: Opioid-induced hyperalgesia
PAH: Pulmonary arterial hypertension
PDGF: Platelet-derived growth factor
PDGFR: Platelet-derived growth factor receptor
RAVE: Relative activity versus endocytosis
RBC: Red blood cell
SCD: Sickle cell disease
SP: Substance P
S1P3R: Sphingosine 1 phosphate receptor 3
Stat3: Signal transducer and activator of transcription 3
TLR4: Toll-like receptor 4
TRV: Tricuspid regurgitant jet velocity
VEGF: Vascular endothelial growth factor
VOC: Vasoocclusive crises.

## References

[1] Piel F. B., Hay S. I., Gupta S., Weatherall D. J., Williams T. N. (2013). Global burden of sickle cell anaemia in children under five, 2010–2050: modelling based on demographics, excess mortality, and interventions. *PLoS Medicine*.

[2] Ballas S. K., Gupta K., Adams-Graves P. (2012). Sickle cell pain: a critical reappraisal. *Blood*.

[3] Fletcher D., Martinez V. (2014). Opioid-induced hyperalgesia in patients after surgery: a systematic review and a meta-analysis. *British Journal of Anaesthesia*.

[4] Kim S. H., Stoicea N., Soghomonyan S., Bergese S. D. (2014). Intraoperative use of remifentanil and opioid induced hyperalgesia/acute opioid tolerance: systematic review. *Frontiers in Pharmacology*.

[5] Soergel D. G., Subach R. A., Burnham N. (2014). Biased agonism of the *μ*-opioid receptor by TRV130 increases analgesia and reduces on-target adverse effects versus morphine: a randomized, double-blind, placebo-controlled, crossover study in healthy volunteers. *Pain*.

[6] Gupta K., Kshirsagar S., Chang L. (2002). Morphine stimulates angiogenesis by activating proangiogenic and survival-promoting signaling and promotes breast tumor growth. *Cancer Research*.

[7] Buchanan I. D., Woodward M., Reed G. W. (2005). Opioid selection during sickle cell pain crisis and its impact on the development of acute chest syndrome. *Pediatric Blood & Cancer*.

[8] Spikes L., Dalvi P., Tawfik O. (2012). Enhanced pulmonary arteriopathy in simian immunodeficiency virus-infected macaques exposed to morphine. *The American Journal of Respiratory and Critical Care Medicine*.

[9] Weber M. L., Farooqui M., Nguyen J. (2008). Morphine induces mesangial cell proliferation and glomerulopathy via *κ*-opioid receptors. *American Journal of Physiology: Renal Physiology*.

[10] Lan X., Rai P., Chandel N. (2013). Morphine induces albuminuria by compromising podocyte integrity. *PLoS ONE*.

[11] Zylla D., Gourley B. L., Vang D. (2013). Opioid requirement, opioid receptor expression, and clinical outcomes in patients with advanced prostate cancer. *Cancer*.

[12] Zylla D., Kuskowski M. A., Gupta K., Gupta P. Association of opioid requirement and cancer pain with survival in advanced non-small cell lung cancer.

[13] Cunningham E. E., Zielezny M. A., Venuto R. C. (1983). Heroin-associated nephropathy. A nationwide problem. *Journal of the American Medical Association*.

[14] Rees D. C., Williams T. N., Gladwin M. T. (2010). Sickle-cell disease. *The Lancet*.

[15] Platt O. S., Brambilla D. J., Rosse W. F. (1994). Mortality in sickle cell disease. Life expectancy and risk factors for early death. *The New England Journal of Medicine*.

[16] Dampier C. D., Smith W. R., Wager C. G. (2013). IMPROVE trial: a randomized controlled trial of patient-controlled analgesia for sickle cell painful episodes: rationale, design challenges, initial experience, and recommendations for future studies. *Clinical Trials*.

[17] (2014). Evidence based management of sickle cell disease. *Expert Panel Report*.

[18] Ballas S. K. (2014). *Sickle Cell Pain*.

[19] Darbari D. S., Neely M., van den Anker J., Rana S. (2011). Increased clearance of morphine in sickle cell disease: implications for pain management. *Journal of Pain*.

[20] Dampier C. D., Setty B. N. Y., Logan J., Ioli J. G., Dean R. (1995). Intravenous morphine pharmacokinetics in pediatric patients with sickle cell disease. *Journal of Pediatrics*.

[21] Kohli D. R., Li Y., Khasabov S. G. (2010). Pain-related behaviors and neurochemical alterations in mice expressing sickle hemoglobin: modulation by cannabinoids. *Blood*.

[22] Hillery C. A., Kerstein P. C., Vilceanu D. (2011). Transient receptor potential vanilloid 1 mediates pain in mice with severe sickle cell disease. *Blood*.

[23] Cain D. M., Vang D., Simone D. A., Hebbel R. P., Gupta K. (2012). Mouse models for studying pain in sickle disease: effects of strain, age, and acuteness. *British Journal of Haematology*.

[24] Vincent L., Vang D., Nguyen J. (2013). Mast cell activation contributes to sickle cell pathobiology and pain in mice.. *Blood*.

[25] Close J., Lottenberg R. Effectiveness of imatinib therapy for a patient with sickle cell anemia and chronic myelocytic leukemia.

[26] Stankovic Stojanovic K., Thiolière B., Garandeau E., Lecomte I., Bachmeyer C., Lionnet F. (2011). Chronic myeloid leukaemia and sickle cell disease: could imatinib prevent vaso-occlusive crisis?. *British Journal of Haematology*.

[27] Biedrzycki O. J., Bevan D., Lucas S. (2009). Fatal overdose due to prescription fentanyl patches in a patient with sickle cell/*β*-thalassemia and acute chest syndrome: a case report and review of the literature. *The American Journal of Forensic Medicine and Pathology*.

[28] Boerner U., Abbott S., Roe R. L. (1975). The metabolism of morphine and heroin in man. *Drug Metabolism Reviews*.

[29] Yeh S. Y. (1975). Urinary excretion of morphine and its metabolites in morphine dependent subjects. *Journal of Pharmacology and Experimental Therapeutics*.

[30] Stain-Texier F., Sandouk P., Scherrmann J.-M. (1998). Intestinal absorption and stability of morphine 6-glucuronide in different physiological compartments of the rat. *Drug Metabolism and Disposition*.

[31] Bodenham A., Quinn K., Park G. R. (1989). Extrahepatic morphine metabolism in man during the anhepatic phase of orthotopic liver transplantation. *British Journal of Anaesthesia*.

[32] Waldhoer M., Bartlett S. E., Whistler J. L. (2004). Opioid receptors. *Annual Review of Biochemistry*.

[33] Whistler J. L., Chuang H.-H., Chu P., Jan L. Y., Von Zastrow M. (1999). Functional dissociation of *μ* opioid receptor signaling and endocytosis: implications for the biology of opiate tolerance and addiction. *Neuron*.

[34] Sternini C., Spann M., Anton B. (1996). Agonist-selective endocytosis of *μ* opioid receptor by neurons in vivo. *Proceedings of the National Academy of Sciences of the United States of America*.

[35] He L., Fong J., von Zastrow M., Whistler J. L. (2002). Regulation of opioid receptor trafficking and morphine tolerance by receptor oligomerization. *Cell*.

[36] Gupta K., Stephenson E. (2007). *The Endothelium: A Comprehensive Reference*.

[37] Wen H., Lu Y., Yao H., Buch S. (2011). Morphine induces expression of platelet-derived growth factor in human brain microvascular endothelial cells: implication for vascular permeability. *PLoS ONE*.

[38] Luk K., Boatman S., Johnson K. N. (2012). Influence of morphine on pericyte-endothelial interaction: implications for antiangiogenic therapy. *Journal of Oncology*.

[39] Singleton P. A., Moreno-Vinasco L., Sammani S., Wanderling S. L., Moss J., Garcia J. G. N. (2007). Attenuation of vascular permeability by methylnaltrexone: role of mOP-R and S1P3 transactivation. *The American Journal of Respiratory Cell and Molecular Biology*.

[40] Singleton P. A., Lingen M. W., Fekete M. J., Garcia J. G. N., Moss J. (2006). Methylnaltrexone inhibits opiate and VEGF-induced angiogenesis: role of receptor transactivation. *Microvascular Research*.

[41] Chen C., Farooqui M., Gupta K. (2006). Morphine stimulates vascular endothelial growth factor-like signaling in mouse retinal endothelial cells. *Current Neurovascular Research*.

[42] Farooqui M., Li Y., Rogers T. (2007). COX-2 inhibitor celecoxib prevents chronic morphine-induced promotion of angiogenesis, tumour growth, metastasis and mortality, without compromising analgesia. *British Journal of Cancer*.

[43] Hatzipantelis E. S., Pana Z. D., Gombakis N. (2013). Endothelial activation and inflammation biomarkers in children and adolescents with sickle cell disease. *International Journal of Hematology*.

[44] Hyacinth H., Gee B., Adamkiewicz T. (2012). Plasma BDNF and PDGF-AA levels are associated with high TCD velocity and stroke in children with sickle cell anemia. *Cytokine*.

[45] Niu X., Nouraie M., Campbell A. (2009). Angiogenic and inflammatory markers of cardiopulmonary changes in children and adolescents with sickle cell disease. *PLoS ONE*.

[46] Wang X., Loram L. C., Ramos K. (2012). Morphine activates neuroinflammation in a manner parallel to endotoxin. *Proceedings of the National Academy of Sciences of the United States of America*.

[47] Hutchinson M. R., Shavit Y., Grace P. M., Rice K. C., Maier S. F., Watkins L. R. (2011). Exploring the neuroimmunopharmacology of opioids: an integrative review of mechanisms of central immune signaling and their implications for opioid analgesia. *Pharmacological Reviews*.

[48] Belcher J. D., Chen C., Nguyen J. (2014). Heme triggers TLR4 signaling leading to endothelial cell activation and vaso-occlusion in murine sickle cell disease. *Blood*.

[49] Ghosh S., Adisa O. A., Chappa P. (2013). Extracellular hemin crisis triggers acute chest syndrome in sickle mice. *Journal of Clinical Investigation*.

[50] Michaels L. A., Ohene-Frempong K., Zhao H., Douglas S. D. (1998). Serum levels of substance P are elevated in patients with sickle cell disease and increase further during vaso-occlusive crisis. *Blood*.

[51] Mcclellan A. C., Luthi J. C., Lynch J. R. (2012). High one year mortality in adults with sickle cell disease and end-stage renal disease. *British Journal of Haematology*.

[52] Scheinman J. I. (2009). Sickle cell disease and the kidney. *Nature Clinical Practice Nephrology*.

[53] Da Silva Junior G. B., Libório A. B., De Francesco Daher E. (2011). New insights on pathophysiology, clinical manifestations, diagnosis, and treatment of sickle cell nephropathy. *Annals of Hematology*.

[54] Arerangaiah R., Chalasani N., Udager A. (2007). Opioids induce renal abnormalities in tumor-bearing mice. *Nephron Experimental Nephrology*.

[55] Weber M. L., Chen C., Li Y. (2013). Morphine stimulates platelet-derived growth factor receptor-*β* signalling in mesangial cells *in vitro* and transgenic sickle mouse kidney *in vivo*. *British Journal of Anaesthesia*.

[56] Atici S., Cinel I., Cinel L., Doruk N., Eskandari G., Oral U. (2005). Liver and kidney toxicity in chronic use of opioids: an experimental long term treatment model. *Journal of Biosciences*.

[57] Borzelleca J. F., Egle J. L., Harris L. S., Johnson D. N., Terrill J. B., Nuite Belleville J. A. (1994). Toxicological evaluation of *μ*-agonists part I: assessment of toxicity following 30 days of repeated oral dosing of male and female rats with levo-alpha-acetylmethadol HCl (LAAM). *Journal of Applied Toxicology*.

[58] Singhal P. C., Pamarthi M., Shah R., Chandra D., Gibbons N. (1994). Morphine stimulates superoxide formation by glomerular mesangial cells. *Inflammation*.

[59] Sharp B. M., Keane W. F., Suh H. J., Gekker G., Tsukayama D., Peterson P. K. (1985). Opioid peptides rapidly stimulate superoxide production by human polymorphonuclear leukocytes and macrophages. *Endocrinology*.

[60] Weber M. L., Vang D., Velho P. E. (2012). Morphine promotes renal pathology in sickle mice. *International Journal of Nephrology and Renovascular Disease*.

[61] Singhal P. C., Pan C. Q., Sagar S., Gibbons N., Valderrama E. (1995). Morphine enhances deposition of ferritin-antiferritin complexes in the glomerular mesangium. *Nephron*.

[62] Kapasi A. A., Gibbons N., Mattana J., Singhal P. C. (2000). Morphine stimulates mesangial cell TNF-*α* and nitrite production. *Inflammation*.

[63] Kilcoyne M. M., Gocke D. J., Meltzer J. I. (1972). Nephrotic syndrome in heroin addicts. *The Lancet*.

[64] Benjamin L., Max M. B., Portenoy R. K., Laska E. M. (1991). Sickle cell disease. *Advances in Pain Research and Therapy*.

[65] Birken C., Khambalia A., Dupuis A. (2013). Morphine is associated with acute chest syndrome in children hospitalized with sickle cell disease. *Hospital Pediatrics*.

[66] Ballas S. K., Park C. H. (1991). Severe hypoxemia secondary to acute sternal infarction in sickle cell anemia. *Journal of Nuclear Medicine*.

[67] Bellet P. S., Kalinyak K. A., Shukla R., Gelfand M. J., Rucknagel D. L. (1995). Incentive spirometry to prevent acute pulmonary complications in sickle cell diseases. *The New England Journal of Medicine*.

[68] Rucknagel D. L., Kalinyak K. A., Gelfand M. J. (1991). Rib infarcts and acute chest syndrome in sickle cell diseases. *The Lancet*.

[69] Vichinsky E. P., Neumayr L. D., Earles A. N. (2000). Causes and outcomes of the acute chest syndrome in sickle cell disease. *The New England Journal of Medicine*.

[70] Styles L. A., Schalkwijk C. G., Aarsman A. J., Vichinsky E. P., Lubin B. H., Kuypers F. A. (1996). Phospholipase A2 levels in acute chest syndrome of sickle cell disease. *Blood*.

[71] Ashcroft M. T., Serjeant G. R. (1981). Growth, morbidity, and mortality in a cohort of Jamaican adolescents with homozygous sicke cell disease. *West Indian Medical Journal*.

[72] Sprinkle R. H., Cole T., Smith S., Buchanan G. R. (1986). Acute chest syndrome in children with sickle cell disease. A retrospective analysis of 100 hospitalized cases. *American Journal of Pediatric Hematology/Oncology*.

[73] Vichinsky E. P., Lubin B. H. (1980). Sickle cell anemia and related hemoglobinopathies. *Pediatric Clinics of North America*.

[74] Kopecky E. A., Jacobson S., Joshi P., Koren G. (2004). Systemic exposure to morphine and the risk of acute chest syndrome in sickle cell disease. *Clinical Pharmacology and Therapeutics*.

[75] Lewing K., Britton K., Debaun M., Woods G. (2011). The impact of parenteral narcotic choice in the development of acute chest syndrome in sickle cell disease. *Journal of Pediatric Hematology/Oncology*.

[76] Kassim A. A., DeBaun M. R. (2013). Sickle cell disease, vasculopathy, and therapeutics. *Annual Review of Medicine*.

[77] Kato G. J., Gladwin M. T., Steinberg M. H. (2007). Deconstructing sickle cell disease: reappraisal of the role of hemolysis in the development of clinical subphenotypes. *Blood Reviews*.

[78] Parent F., Bachir D., Inamo J. (2011). A hemodynamic study of pulmonary hypertension in sickle cell disease. *The New England Journal of Medicine*.

[79] Dham N., Ensing G., Minniti C. (2009). Prospective echocardiography assessment of pulmonary hypertension and its potential etiologies in children with sickle cell disease. *The American Journal of Cardiology*.

[80] Farmakis D., Aessopos A. (2011). Pulmonary hypertension associated with hemoglobinopathies: prevalent but overlooked. *Circulation*.

[81] Gladwin M. T., Vichinsky E. (2008). Pulmonary complications of sickle cell disease. *The New England Journal of Medicine*.

[82] Nguyen J., Luk K., Vang D. (2014). Morphine stimulates cancer progression and mast cell activation and impairs survival in transgenic mice with breast cancer. *British Journal of Anaesthesia*.

[83] Farha S., Sharp J., Asosingh K. (2012). Mast cell number, phenotype, and function in human pulmonary arterial hypertension. *Pulmonary Circulation*.

[84] Lee C. W.-S., Muo C.-H., Liang J.-A., Sung F.-C., Kao C.-H. (2013). Association of intensive morphine treatment and increased stroke incidence in prostate cancer patients: a population-based nested case-control study. *Japanese Journal of Clinical Oncology*.

[85] Koch J., Manworren R., Clark L., Quinn C. T., Buchanan G. R., Rogers Z. R. (2008). Pilot study of continuous co-infusion of morphine and naloxone in children with sickle cell pain crisis. *American Journal of Hematology*.

[86] Mucci-LoRusso P., Berman B. S., Silberstein P. T. (1998). Controlled-release oxycodone compared with controlled-release morphine in the treatment of cancer pain: a randomized, double-blind, parallel-group study. *European Journal of Pain*.

[87] Klepstad P., Borchgrevink P. C., Kaasa S. (2000). Effects on cancer patients’ health-related quality of life after the start of morphine therapy. *Journal of Pain and Symptom Management*.

[88] Panchal S. J., Müller-Schwefe P., Wurzelmann J. I. (2007). Opioid-induced bowel dysfunction: prevalence, pathophysiology and burden. *International Journal of Clinical Practice*.

[89] Portenoy R. K., Thomas J., Moehl Boatwright M. L. (2008). Subcutaneous methylnaltrexone for the treatment of opioid-induced constipation in patients with advanced illness: a double-blind, randomized, parallel group, dose-ranging study. *Journal of Pain and Symptom Management*.

[90] Pizz L. T., Toner R., Foley K. (2012). Relationship between potential opioid-related adverse effects and hospital length of stay in patients receiving opioids after orthopedic surgery. *Pharmacotherapy*.

[91] Wong J. Y., Carvalho B., Riley E. T. (2013). Intrathecal morphine 100 and 200 *μ*g for post-cesarean delivery analgesia: a trade-off between analgesic efficacy and side effects. *International Journal of Obstetric Anesthesia*.

[92] Nie X., Wen Z.-Y., Yan Z.-Y., Huang L., Sun D., Cheng B. (2000). Effects of morphine on rheological properties of rat red blood cells. *Clinical Hemorheology and Microcirculation*.

[93] Schein A., Enriquez C., Coates T. D., Wood J. C. (2008). Magnetic resonance detection of kidney iron deposition in sickle cell disease: a marker of chronic hemolysis. *Journal of Magnetic Resonance Imaging*.

[94] Savov Y., Antonova N., Zvetkova E., Gluhcheva Y., Ivanov I., Sainova I. (2006). Whole blood viscosity and erythrocyte hematometric indices in chronic heroin addicts. *Clinical Hemorheology and Microcirculation*.

[95] Brown L. S., Hickson M. J., Ajuluchukwu D. C., Bailey J. (1993). Medical disorders in a cohort of New York City drug abusers: Much more than HIV disease. *Journal of Addictive Diseases*.

[96] Zeiger A. R., Patkar A. A., Fitzgerald R., Lundy A., Ballas S. K., Weinstein S. P. (2002). Changes in mu opioid receptors and rheological properties of erythrocytes among opioid abusers. *Addiction Biology*.

[97] Senturk M., Kufrevioglu O. I., Ciftci M. (2009). Effects of some analgesic anaesthetic drugs on human erythrocyte glutathione reductase: an in vitro study. *Journal of Enzyme Inhibition and Medicinal Chemistry*.

[98] Halabi-Tawil M., Lionnet F., Girot R., Bachmeyer C., Lévy P. P., Aractingi S. (2008). Sickle cell leg ulcers: a frequently disabling complication and a marker of severity. *British Journal of Dermatology*.

[99] Connes P., Lamarre Y., Hardy-Dessources M.-D. (2013). Decreased hematocrit-to-viscosity ratio and increased lactate dehydrogenase level in patients with sickle cell anemia and recurrent leg ulcers. *PLoS ONE*.

[100] Madu A. J., Ubesie A., Madu K. A., Okwor B., Anigbo C. (2013). Evaluation of clinical and laboratory correlates of sickle leg ulcers. *Wound Repair and Regeneration*.

[101] Minniti C. P., Delaney K.-M. H., Gorbach A. M. (2014). Vasculopathy, inflammation, and blood flow in leg ulcers of patients with sickle cell anemia. *American Journal of Hematology*.

[102] Queiroz A. M. M., Campos J., Lobo C., Bonini-Domingos C. R., Cardoso G., Ballas S. K. (2014). Leg amputation for an extensive, severe and intractable sickle cell anemia ulcer in a Brazilian patient. *Hemoglobin*.

[103] Ballas S. K. (2002). Treatment of painful sickle cell leg ulcers with topical opioids. *Blood*.

[104] Poonawala T., Levay-Young B. K., Hebbel R. P., Gupta K. (2005). Opioids heal ischemic wounds in the rat. *Wound Repair and Regeneration*.

[105] Gupta M., Poonawala T., Farooqui M., Ericson M., Gupta K. (2014). Topical fentanyl stimulates healing of ischaemic wounds in diabetic rats. *Journal of Diabetes*.

[106] Farley P. (2011). Should topical opioid analgesics be regarded as effective and safe when applied to chronic cutaneous lesions?. *Journal of Pharmacy and Pharmacology*.

[107] Goldsmith J. R., Uronis J. M., Jobin C. (2011). Mu opioid signaling protects against acute murine intestinal injury in a manner involving Stat3 signaling. *The American Journal of Pathology*.

[108] Bigliardi-Qi M., Gaveriaux-Ruff C., Pfaltz K. (2007). Deletion of *μ*- and *κ*-opioid receptors in mice changes epidermal hypertrophy, density of peripheral nerve endings, and itch behavior. *Journal of Investigative Dermatology*.

[109] Sim R., Cheong D. M., Wong K. S., Lee B. M. K., Liew Q. Y. (2007). Prospective randomized, double-blind, placebo-controlled study of pre- and postoperative administration of a COX-2-specific inhibitor as opioid-sparing analgesia in major colorectal surgery. *Colorectal Disease*.

[110] Moss J., Rosow C. E. (2008). Development of peripheral opioid antagonists: new insights into opioid effects. *Mayo Clinic Proceedings*.

